# Infectious complications after anti-BCMA CAR-T cell therapy in patients with multiple myeloma: a systematic literature review

**DOI:** 10.1017/ash.2026.10830

**Published:** 2026-09-09

**Authors:** Arthur Franco Cotia, André Luís Franco Cotia, Beatriz Bonini Zancopé, Newton de Freitas Centurião, Mariana Kim Hsieh, Erika Viana-Cardenas, Patricia Deffune Celeghini, Pietra Sovero, Isabele Pardo, Vinicius S. Marra, Carlos Henrique Spadão Tudino, Jorge L. Salinas, Alex N. Zimmet, Pedro Hsieh, Nelson Hamerschlak, Mariana Nassif Kerbauy, Michael B. Edmond, Alexandre R. Marra

**Affiliations:** 1 Universidade Federal de São Paulo (UNIFESP), São Paulo, SP, Brazil; 2 Hospital Israelita Albert Einstein, São Paulo, SP, Brazil; 3 Faculdade Israelita de Ciências da Saúde Albert Einstein, Hospital Israelita Albert Einstein, São Paulo, SP, Brazil; 4 Program of Hospital Epidemiology, University of Iowa Health Care, Iowa City, IA, USA; 5 Division of Infectious Diseases & Geographic Medicine, Stanford University School of Medicine, Standford, CA, USA; 6 University of Iowa Health Care, Iowa City, IA, USA; 7 Department of Medicine, West Virginia University School of Medicine, Morgantown, WV, USA

## Abstract

**Background:**

Chimeric antigen receptor T-cell (CAR-T) therapy has transformed the treatment of relapsed/refractory multiple myeloma but is associated with significant infectious complications due to prolonged immunosuppression, cytopenias, and immune dysfunction.

**Objective:**

To systematically evaluate the incidence, timing, microbiologic patterns, and risk factors of infectious complications in patients with multiple myeloma undergoing anti-BCMA CAR-T cell therapy.

**Study design:**

Systematic review following PRISMA guidelines. We searched PubMed, EMBASE, Scopus, CINAHL, Cochrane Library, and Google Scholar through February 2025. Eligible studies included cohorts, phase II and III trials, and case-control studies reporting infections after CAR-T therapy. Data were extracted independently and quality assessed with the Downs and Black scale. Owing to heterogeneity, results were synthesized descriptively. Etiology and temporal patterns were analyzed at the event level.

**Results:**

Nineteen studies published between 2021 and 2025, including 1,495 patients with multiple myeloma treated with CAR T-cell therapy, were included. Infection incidence ranged from approximately 33% to over 70%. Bacterial infections predominated, particularly during the first 30 days after infusion, whereas viral infections increased during later follow-up. Fungal infections were less frequent. Rates of severe infection varied widely (5% to 60%), and infection-related mortality ranged from 0% to over 5%. Key risk factors included prolonged cytopenias, low CD4^+^ T-cell, and hypogammaglobulinemia.

**Conclusions:**

Infectious complications following CAR-T cell therapy in multiple myeloma are frequent, with distinct early and late microbiologic profiles. Standardized infection definitions, harmonized surveillance, and risk-stratified prevention strategies are essential to reduce infection-related morbidity while preserving the benefits of this therapy.

## Graphical abstract

**Figure:**
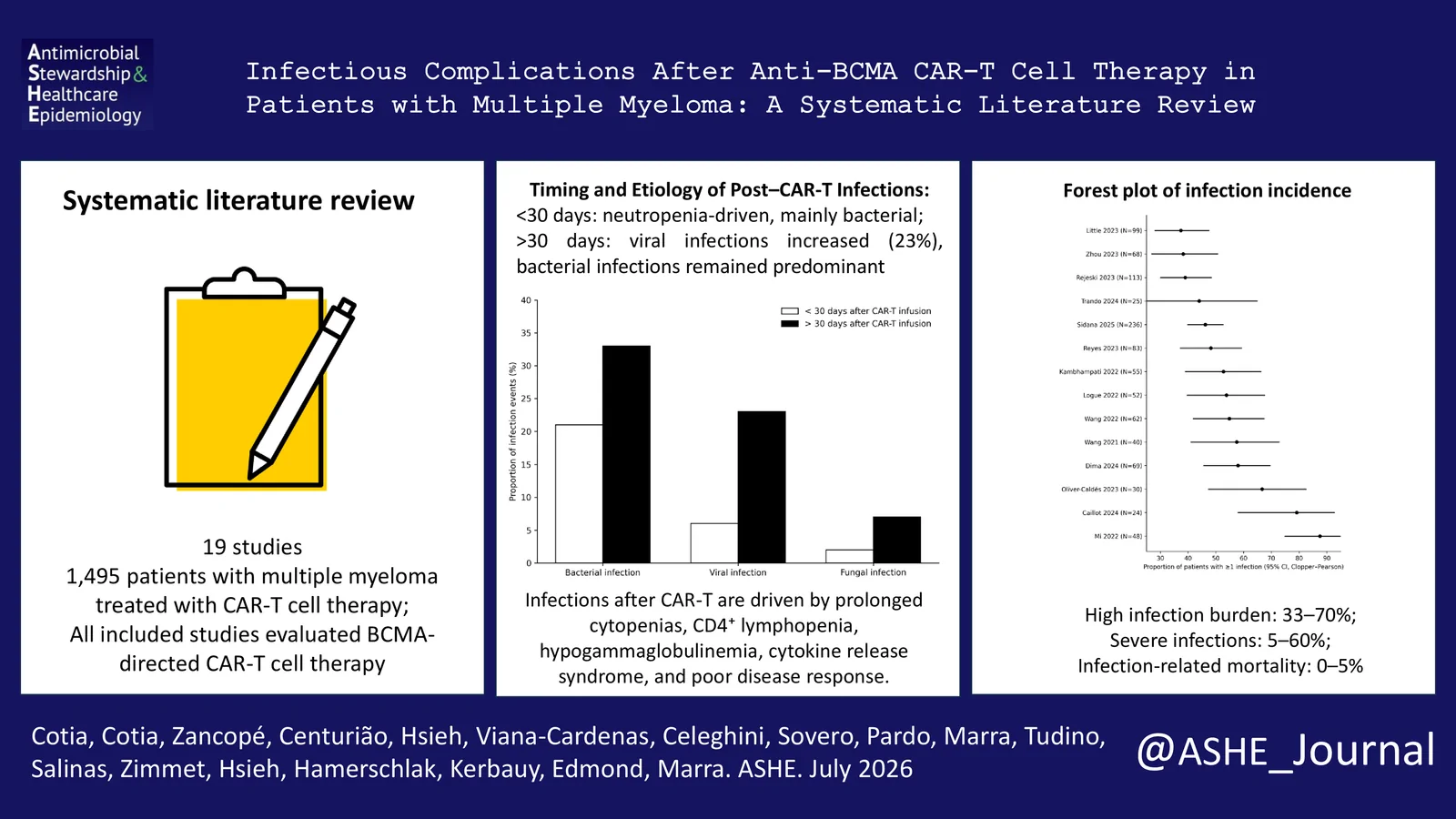

## Introduction

Chimeric antigen receptor T-cell (CAR-T) therapy is an advanced form of immunotherapy where T lymphocytes are genetically engineered to recognize and eliminate tumor cells. This modification enables T cells to express a synthetic receptor capable of identifying specific antigens on malignant cells, promoting a targeted immune response. Since its introduction, CAR-T cell therapy has been used in treating hematologic malignancies, including refractory or relapsed B-cell leukemias and lymphomas, demonstrating significant remission rates.^1,2^

Multiple myeloma, characterized by clonal plasma cell proliferation in the bone marrow, remains challenging. Despite advances with immunomodulatory drugs, proteasome inhibitors, and anti-CD38 monoclonal antibodies, prognosis for relapsed or refractory disease is poor. In this context, B cell maturation antigen (BCMA)-directed CAR-T cell therapies have emerged as an innovative and highly effective option, with overall response rates of 70% to 95% in clinical trials. Idecabtagene vicleucel and ciltacabtagene autoleucel, are notable examples.^3^

CAR T-cell therapy involves ex vivo genetic modification and expansion of autologous T lymphocytes, followed by reinfusion after lymphodepleting chemotherapy. While transformative for multiple myeloma, this approach is associated with serious adverse events, including cytokine release syndrome (CRS), neurotoxicity, and infectious complications.^4^ Prolonged immunosuppression, cytopenias, and B-cell aplasia render patients vulnerable to various pathogens. Despite the clinical severity of posttreatment infections, recent systematic reviews comprehensively addressing their incidence and risk factors in multiple myeloma patients are lacking.^1,5–7^

This study aims to systematically review the literature to determine the overall incidence of infections in multiple myeloma patients undergoing CAR T-cell therapy, identify common infection types and examine their timing relative to the CAR-T therapeutic course. It also seeks to evaluate both patient- and treatment-related factors, providing a comprehensive assessment of infectious complications associated with this approach.

## Methods

### Systematic literature review

This review followed the PRISMA (Preferred Reporting Items for Systematic Reviews and Meta-Analyses) methodology.^8^ It was registered on PROSPERO on January 20th, 2025 (CRD42025640975). Institutional approval was not required.

### Search strategy

A comprehensive literature search was performed in PubMed, Embase, Scopus, CINAHL, Cochrane, and Google Scholar, for studies published up to February 2025 reporting infectious outcomes in patients with multiple myeloma treated with CAR-T therapy. The search strategy used controlled vocabulary and free-text terms related to hematological malignancies, CAR-T therapy, and infections. No language restrictions were applied; only full-text articles were eligible. Reference lists were manually screened. Detailed search strategies are in Supplementary Material.

### Inclusion criteria

Studies were eligible if they included adult or pediatric multiple myeloma patients receiving CAR-T therapy (BCMA-directed or other strategies) and reported infectious outcomes, including incidence, pathogen identification, timing, severity, or risk factors. Eligible designs were phase 2 and phase 3 clinical trials, cohort studies, and case-control studies.

### Exclusion criteria

Studies with <20 participants; case reports; phase 1 clinical trials; non-human studies; review articles; conference abstracts; and studies without infection outcomes; studies focused exclusively on non-infectious complications; and studies of anti-CD19 CAR-T therapy without a BCMA-directed component were excluded.

### Outcomes

Primary outcomes included: (1) overall infection incidence; (2) infection types (bacterial, viral, fungal, and parasitic); (3) timing relative to CAR-T infusion; (4) risk factors.

Secondary outcomes included: infection severity, assessed using Common Terminology Criteria for Adverse Events (CTCAE), version 5.0 criteria,^9^ or other criteria; pathogen profiles (including multidrug-resistant organisms); antimicrobial use; infection-related complications.

### Study selection and data extraction

Titles and abstracts were screened using Rayyan^10^ followed by full-text evaluation, conducted independently by two trained investigators (A.L.F.C. and A.F.C.). Data extraction from included studies was performed by two of nine independent investigators (B.B.Z., C.H.S.T., E.V.C., I.P., M.K.H., N.F.C., P.D.C., P.S.C., V.S.M.), using a standardized data abstraction form (Supplementary Appendix 2). Discrepancies were resolved by discussion or a third reviewer.

Extracted variables included study characteristics, patient characteristics, infectious outcomes, timing, risk factors, antimicrobial therapies, and outcomes.

**Figure 1.**
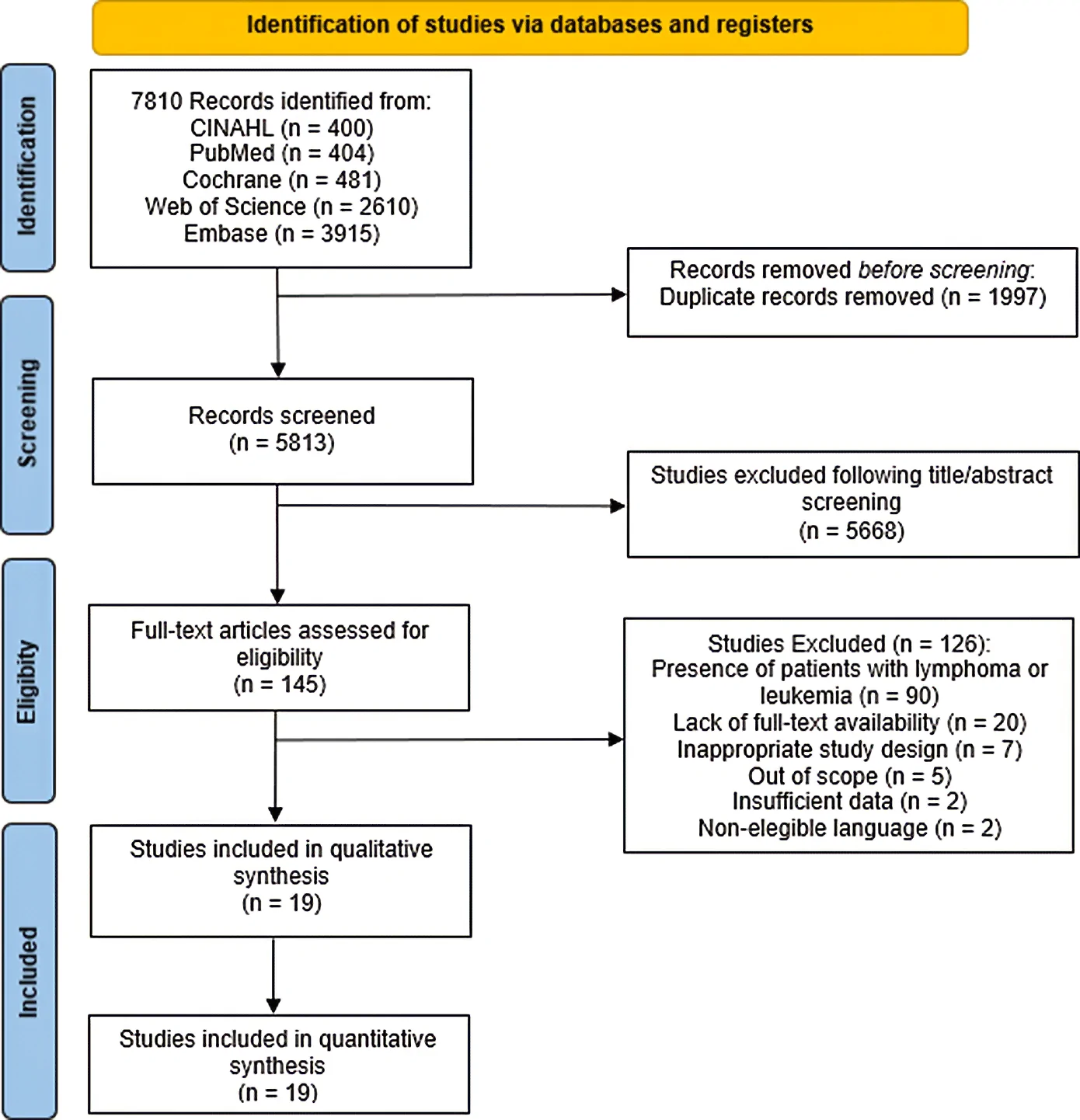
Literature search for articles evaluating infectious complications after CAR T-cell therapy in patients with multiple myeloma.

Study selection followed PRISMA guidelines (Figure 1).

### Quality assessment and risk of bias

Methodological quality was assessed using the Downs and Black scale,^11^ a validated tool for assessing the methodological quality and risk of bias in randomized and non-randomized studies, by two independent reviewers, with discrepancies resolved by third reviewer. Question 27 was modified to a yes/no format (maximum score 28). Scores were categorized as good (≥18), fair (14–17), or poor (<14).

### Statistical analysis and evidence synthesis

Due to heterogeneity, a formal meta-analysis was not performed. Data were synthesized descriptively.

Infection incidence was calculated as the proportion of patients experiencing at least one infectious case, with 95% confidence intervals estimated using exact binomial (Clopper–Pearson) methods. Infection-related mortality rates were summarized with 95% confidence intervals using the Wilson method. Forest plots illustrated variability without statistical pooling.

Infection etiology was analyzed at the event level. Proportions of bacterial, viral, and fungal involvement were extracted; categories were not mutually exclusive and therefore did not sum to 100%.

Temporal patterns used study-specific definitions (commonly a 30-day threshold). Severe infection rates and effect estimates (ORs, HRs, and RRs) were summarized.

### Patient consent statement

As a systematic review of published deidentified data, no patient consent was required.

## Results

The search identified 7,810 records. After screening, 19 studies were included in the qualitative synthesis (Figure 1).

These studies, published between 2021 and 2025 comprised 1,495 patients with relapsed or refractory multiple myeloma treated with CAR T-cell therapy. Most studies were retrospective cohort analyses^12–27^ (16 of 19 studies), frequently based on real-world or early-access program data. The remaining studies included two phase II trials^28,29^ and one quasi-experimental study.^30^ Most cohorts originated from the United States^12,14–16,18,21,22,24,26,27^ (n = 10) and China^13,17,19,28,29^ (n = 5), with additional contributions from Spain,^30^ France,^23^ Germany,^25^ and one international multicenter consortium.^20^

Patients were consistently heavily pretreated, with a median of 4 to 6 prior lines of therapy, frequently including immunomodulatory agents, proteasome inhibitors, and anti-CD38 monoclonal antibodies. Triple-class refractory disease was common, approximately 89% of patients met this criterion. Prior autologous stem cell transplantation was reported in approximately 80% to 100% of patients, whereas prior allogeneic transplantation was rare.^21^ Median age ranged from the mid-50s to mid-60s, and the follow-up duration varied from approximately 3 months to 36 months.^12–30^

Most studies evaluated commercially available BCMA-directed CAR T-cell products, predominantly idecabtagene vicleucel (12 studies) and ciltacabtagene autoleucel (9 studies). A small number of cohorts included investigational BCMA constructs, dual-targeting approaches, or institutionally developed CAR-T products.^17,19,30^ One study compared infectious outcomes across BCMA-directed and non–BCMA-directed CAR-T therapies and bispecific antibodies in patients with prior allogeneic transplantation.^21^ Infection outcomes were evaluated in most studies, although definitions, diagnostic criteria, and follow-up windows varied. Four studies explicitly defined microbiologically confirmed infections while also accepting clinically diagnosed infections.^14,15,17,19^ Infection severity was graded using the Common Terminology Criteria for Adverse Events (CTCAE), version 5.0^9^ in most cohorts (14 studies),^13–16,18,19,21–23,25–30^ while four studies^12,17,20,24^ applied institutional or alternative grading criteria. One study^16^ did not report a formal severity grading. Across the included studies, some cohorts reported specific infectious etiologies or syndrome-level diagnoses,^14,17,18,23,24,30^ whereas others provided broader categorizations by pathogen class, including bacterial, viral, fungal, and, more rarely, parasitic infections.^13,16,19–21,26,28^ Several cohorts further classified infections by anatomic site, including lower and upper respiratory tract infections, bloodstream infections, gastrointestinal infections, genitourinary infections, and skin or soft tissue involvement.^13–15,18,19,24,27–29^

Immune effector cell–related toxicities and their management were also explored as potential predictors, including CRS, Immune Effector Cell-Associated Neurotoxicity Syndrome (ICANS), and the use of immunosuppressive therapies such as corticosteroids or tocilizumab; however, these variables were not consistently associated with infection risk.^18^ Across studies, impaired immune recovery and hematologic toxicity were consistently associated with higher infection rates and infection-related mortality.^13,17–20,24–26^ Patients with high CAR-HEMATOTOX scores had an eightfold higher rate of severe infections (40% vs 5%) and higher 1-year non-relapse mortality (13% vs 2%),^20^ while hypogammaglobulinemia independently predicted late respiratory infections (HR 6.06).^18^ Similarly, elevated baseline EASIX scores were associated with severe late-onset infections and impaired hematopoietic recovery.^25^ Supportive care and prophylactic strategies varied widely across studies.^13–15,17–20,22,24,28^ Although antiviral prophylaxis was commonly reported across studies, several cohorts explicitly described the use of acyclovir or valacyclovir–based antiviral prophylaxis consistent with herpes simplex virus and varicella–zoster virus (HSV/VZV) prevention strategies.^14,15,17–20,25^ In contrast, cytomegalovirus (CMV)-specific prophylaxis was not routinely reported, and CMV monitoring strategies were rarely detailed. While CMV reactivation or CMV DNAemia was documented in several cohorts,^23,24,27^ reporting of CMV prevention approaches remained heterogeneous, precluding conclusions regarding routine CMV prophylaxis practices. *Pneumocystis jirovecii* pneumonia prophylaxis was routinely administered, with no confirmed cases of Pneumocystis infection reported. Antibacterial and antifungal prophylaxis were generally restricted to periods of neutropenia or corticosteroid exposure. Many cohorts reported intravenous immunoglobulin (IVIG) replacement for hypogammaglobulinemia^13–15,17–20,26–28,30^ and granulocyte colony-stimulating factor (G-CSF) support,^13–15,18–20,24–27^ with variability in initiation timing, and duration. These differences likely contributed to heterogeneity in infection incidence across studies (Table 1). Considerable heterogeneity was observed in how infectious outcomes were reported across studies, including differences in the definition of early and late infection periods, reporting of infection severity, etiologic classification, and infection-related mortality (Table 2).

**Table 1.** Characteristics of included studies evaluating infectious complications after CAR T-cell therapy in multiple myeloma Table 1 long description.

First author, year (country)/Study design	Sample size (n)/Follow-up duration	Median age, years/Male, n (%)	CAR-T product/target	Prior lines of therapy/Prior HSCT, n (%)	Anti-infective prophylaxis	Infection outcomes	Key infection findings
Banerjee, 2021 (USA)/retrospective cohort	50/15.3 months	NR/27 (54)	Idecabtagene vicleucel, Ciltacabtagene autoleucel, Orvacabtagene autoleucel, BB21217 (anti-BCMA)	>3 prior lines/NR	NR	Severe infections requiring IV antibiotics; association with CRS timing and early vs late tocilizumab	Severe infections in 6% of patients; similar rates in early vs late tocilizumab groups; early tocilizumab did not increase infection risk
Wang, 2021 (China)/retrospective cohort	40/16 months	55/27 (67.5)	Anti-BCMA CAR-T (CD28 or 4–1BB)	Median 4 (range 2–9)/NR	Antibacterial/antifungal prophylaxis; IVIG for IgG < 400 mg/dL in patients with infections	Microbiologically confirmed and clinically diagnosed infections; severity (mild to fatal); timing and type (bacterial, viral, fungal); site and correlation with immunoglobulin levels	Infections in 57.5% (44 events); 47.7% severe; no infection-related deaths. Bacterial 57%, viral 18%, fungal 7%; lungs most frequently (52%); 43% within 60 days. Lower IgG associated with severe infections
Kambhampati, 2022 (USA)/retrospective cohort	55/Median 6 months	62/27 (49)	Ciltacabtagene autoleucel, Idecabtagene vicleucel, BB21217, JCARH125 (anti-BCMA)	Median 6 lines (range 1–13); 89% triple-class refractory/48 (87)	Antiviral: VZV prophylaxis; antibacterial and antifungal prophylaxis during ANC < 500 cells/µL; PJP prophylaxis for CD4 < 200 cells/µL; IVIG 400 mg/kg for IgG < 400 mg/dL	Bacterial, viral, and fungal infections up to 1 year; organ system, severity, timing, and impact on OS/PFS	Infections in 53% (40% bacterial, 53% viral, 6% fungal), mostly mild–moderate and respiratory. Half within 100 days; one fatal Aspergillus pneumonia. No clear risk factors or impact on survival
Logue, 2022 (USA)/retrospective cohort	52/100 days	66/23 (44)	Idecabtagene vicleucel	4 prior lines; 87% triple-class refractory/81% autologous; no allogeneic	Antiviral: acyclovir; antibacterial: levofloxacin; antifungal: fluconazole or posaconazole during neutropenia; PJP prophylaxis: TMP-SMX (day 30–180)	Clinically and microbiologically diagnosed infections within 100 days; CTCAE-graded; early (≤30 days) vs late (31–100 days); pathogen type	Infections in 54% (23% grade ≥3). Early infections are mostly bacterial (68%, 50% severe). Late infections: 50% bacterial (mainly sinusitis), 42% viral (mostly COVID-19). No infection-related deaths. Longer interval between bridging therapy and lymphodepletion associated with higher infection risk
Mi, 2022 (China)/phase II, open-label trial	48/Median 18 months	61/32 (66.7)	Ciltacabtagene autoleucel	Median 4 (range 3–9)/17 (35.4)	Antiviral prophylaxis in 24/48 patients (regimen not specified)	All-grade and grade 3/4 infections (pneumonia, URTI, herpes zoster, HBV reactivation/new infection); timing post infusion	Infections in 85.4% (37.5% grade 3/4). Most common: pneumonia (35.4%), URTI (33.3%), herpes zoster (18.8%). HBV reactivation 10.4%, new HBV 6.3%; no COVID-19
Wang, 2022 (China)/phase II, single-arm trial	62/Median 21.3 months	58/34 (55)	Ciltacabtagene autoleucel	Median 4 (range 2–17)/17 (27)	NR	Incidence, timing, type (bacterial, viral, fungal), and CTCAE ≥3 infections within 100 days; association with cytopenias and immune reconstitution	Infections in 54%; 23% grade ≥3. Early (≤30 days) infections predominantly bacterial (68%, 50% severe). Late (31–100 days) infections: 50% bacterial, 42% viral; fungal infections rare. Longer interval from last bridging therapy associated with higher infection risk
Cheng, 2023 (China)/retrospective cohort	126/24 months	58/68 (54)	Single-target anti-BCMA (n = 28), anti-BCMA + anti-CD19 (n = 54), tandem anti-BCMA + humanized anti-CD19 (n = 44)	Median 4 (2–16) prior lines; 6% prior CAR-T/39 (31) prior ASCT	Antiviral: acyclovir 400 mg BID from lymphodepletion to ≥3 months; antifungal: posaconazole 200 mg TID during ANC < 500; PJP: TMP-SMX 800 mg BID 2 days/week for ≥3 months; no routine antibacterial prophylaxis	Incidence, timing (early ≤30 days vs late >30 days), severity, site, and microbiologic type; association with CD4^+^ T-cell recovery and predictive model	Infections in 71/126 (56%), 85 events (29 early, 56 late). Early infections predominantly bacterial pneumonia/URTI; most mild–moderate. Late infections more often severe (36%), mostly bacterial (80%). One death from bacteremia. CD4^+^ count at day 30 lower in patients who later developed infection; predictive model AUROC 0.63
Hammons, 2023 (USA)/retrospective cohort	33 (24 CAR-T, 9 BsAb)/CAR-T: 8.5 months; BsAb: 11 months	CAR-T: 60; BsAb: 65/CAR-T: 12 (50); BsAb: 3 (34)	Ciltacabtagene autoleucel, Idecabtagene vicleucel (CAR-T); various bispecific antibodies	6 prior lines; PI, IMiD, anti-CD38 in almost all/All 24 CAR-T and 9 BsAb had prior allo-HCT (100%)	NR	Grade ≥3 bacterial, viral, fungal infections; correlation with CRS, ICANS, GVHD; infection-related mortality	Grade ≥3 infections in 12/24 (50%) CAR-T and 4/9 (44%) BsAb; mainly bacterial. One fatal infection (Pseudomonas sepsis with BK viremia) in CAR-T patient. No fungal infections; no GVHD exacerbation after CAR-T
Little, 2023 (USA)/retrospective cohort	99/12 months	63/62 (63)	Ciltacabtagene autoleucel, Idecabtagene vicleucel, investigational BCMA CAR-T	>5 prior lines/67 (68)	Antiviral: acyclovir or valacyclovir for 1 year; antibacterial: levofloxacin or ciprofloxacin until count recovery; antifungal: fluconazole during prolonged neutropenia or corticosteroid; PJP: TMP-SMX or atovaquone (day 0–180); IVIG when IgG ≤ 400 mg/dL	Types, timing, severity, and risk factors for infections within 1 year; early (≤100 days) vs late (101–365 days); site and etiology	Infections in 37/99 (37%) with 64 events (42 early, 22 late). Early infections (median day 18) were 69% bacterial, 17% viral, 3% fungal. Late infections (median day 171) were predominantly viral respiratory; 1-year cumulative incidence of respiratory infections 22%. Risk factors for late respiratory infection: hypogammaglobulinemia (IgG ≤ 400 mg/dL) and prior early respiratory viral infection
Oliver-Caldés, 2023 (Spain)/Quasi-experimental	35 (30 evaluable for infections)/36 months	61/18 (35)	ARI0002h (experimental anti-BCMA, 4–1BB)	≥2 lines including PI, IMiD, anti-CD38/28 (93)	IVIG according to institutional guidelines for IgG < 400 mg/dL	Incidence, type, and severity of infections up to 1 year; site (respiratory, systemic, other); pathogen identification	Infections in 20/30 patients (67%), with 45 cases; 16% grade ≥3. Respiratory infections most common (53%), including 3 SARS-CoV-2 cases; one HBV reactivation and one leishmaniasis. One COVID-19–related death
Rejeski, 2023 (France, Germany, USA)/retrospective cohort (multicenter)	113/Median 7.9 months	65/66 (58)	Ciltacabtagene autoleucel, Idecabtagene vicleucel	Median 6 prior lines; prior BCMA therapy and multiple alkylator exposures common/99 (88) prior ASCT; 4 (3.5) prior allo-SCT	Antiviral:acyclovir or valacyclovir; antibacterial: levofloxacin;antifungal: fluconazole, posaconazole,or voriconazoleduring neutropenia (institution-dependent); PJP prophylaxis: TMP-SMX or alternatives until CD4 > 200 cells/µL	Early (≤90 days) infections; type, site, and severity; association between CAR-HEMATOTOX (HT) score and infection risk	Fifty-one infections in 44/113 (39%). Main sites: bloodstream (28%), respiratory (34%), GI (14%). Severe infections more frequent in HT^high vs HT^low (40% vs 5%); 3 infection-related deaths (1 fungal, 2 bacterial). HT score independently predicted severe infection; most NRM events were infection-related
Reyes, 2023 (USA)/retrospective cohort	83/6 months	65/47 (56.7)	Anti-BCMA CAR-T (not specified)	Median 6 prior lines (younger and older groups), including ASCT, IMiD, PI, anti-CD38/68 (81.9)	NR	Confirmed infections within 6 months; incidence and type (bacterial, viral, fungal); comparison ≥70 vs <70 years; association with hypogammaglobulinemia, neutrophil recovery, PFS/OS	Infections in 36% of ≥70 vs 52% of <70 (NS). Viral infections predominated (38% vs 32%), bacterial 20% vs 5%, fungal 5% vs 0%. Most infections mild; no age-related difference in rate, severity, or survival impact
Zhou, 2023 (China)/retrospective cohort	68/20.5 months	45 (66.2)	Anti-BCMA ± anti-CD19 CAR-T	Median 4 prior lines (IQR 2–6)/14 (20.6)	Antiviral: acyclovir 400 mg BID from lymphodepletion to ≥3 months post CAR-T (88.3%); antifungal: azole prophylaxis during ANC <500 (35.3%); PJP: TMP-SMX 800 mg BID 2 days/week (36.8%); IVIG 400 mg/kg for IgG ≤ 400 mg/dL (51.7% of hypogammaglobulinemic patients)	Incidence, timing, type, site, and severity of bacterial, viral, and fungal infections; microbiologic etiology; infection-free survival; risk factors	Forty-three infection events in 26/68 patients (81% bacterial, 12% fungal, 7% viral), mainly pneumonia and bloodstream infections. Severe/life-threatening infections in 79%; 2 infection-related deaths. Independent risk factors: ≥4 prior lines, prolonged neutropenia, high-grade CRS, poor response. Gram-negative bacilli predominated (60.5%)
Caillot, 2024 (France)/retrospective cohort	24/Median 15.2 months	62/11 (45.8)	Idecabtagene vicleucel	IMiD, PI, anti-CD38 in all/24 (100) prior ASCT	IVIG in 21/24 (88%) for hypogammaglobulinemia	Incidence, type, timing, and severity of bacterial, viral, fungal infections; association with hypogammaglobulinemia, cytopenias, CAR-T expansion, PFS/OS	Infections in 19/24 (79%). Viral infections predominant (79%), mainly BK cystitis (29%), COVID-19 (25%), CMV viremia (17%), influenza (8%), mostly grade 1–2. Bacterial infections in 21% (all grade 3; mainly catheter-related Staphylococcus and Pseudomonas colitis). Fungal infections 12.5% (pulmonary aspergillosis, cutaneous mucormycosis). No infection-related deaths
Dima, 2024 (USA)/retrospective cohort	69/10 months	64/34 (49)	Idecabtagene vicleucel (standard-of-care)	6 prior lines; PI, IMiD, anti-CD38/5 (7)	NR	Incidence, timing (≤8 vs >8 weeks), and grade ≥3 infections; comparison with KarMMa-1 trial; association with cytopenias, CRS/ICANS, outcomes	Infections in 40/69 (58%) vs 69% in KarMMa-1; severe infections in 30%. Early infections (≤8 weeks) in 42%. High rates of grade ≥3 neutropenia (87%), anemia (51%), thrombocytopenia (65%). No excess infection-related mortality; most deaths due to disease progression
Frenking, 2024 (Germany)/retrospective cohort	129 (113 evaluable for late infections)/12 months (up to 1 year or relapse/progression)	64/87 (67)	Idecabtagene vicleucel	Median 5 (range 5–7)/NR	Antiviral: acyclovir 400 mg BID until CD4^+^ recovery; antibacterial: rifaximin 200 mg BID until neutrophil recovery; antifungal: fluconazole 200 mg/day or posaconazole 300 mg/day during neutropenia/high risk; PJP prophylaxis: TMP-SMX; IVIG for infection tendency and IgG <4 g/L	All infections from infusion to 1 year or relapse; microbiologically/histopathologically confirmed or treated with antimicrobials; severity classification; association with EASIX and hematologic recovery	Severe late-onset infections (grade ≥3) in 15/113 (13%), mostly bacterial or viral. High baseline EASIX (> upper quartile) associated with higher infection risk (32% vs 8%; OR 5.45). Late infections correlated with prolonged cytopenias and aplastic recovery. No infection-related deaths
Trando, 2024 (USA)/retrospective cohort	25/13 months	65/12 (48)	Idecabtagene vicleucel	Median 6 (range 4–12)/25 (100)	NR	Incidence, type, severity, and site of infections within 6 months; CMV reactivation among CMV-seropositive patients; hypogammaglobulinemia	Infections in 11/25 (44%), 15 events. Respiratory infections predominant (n = 8), followed by GI (n = 2, including C. difficile), bacteremia (n = 2), UTI (n = 2), and one sepsis. Severe infections in 3 patients (12%). CMV reactivation in 9/19 CMV-seropositive (47%), all mild. Hypogammaglobulinemia in 96%; 80% required IVIG. No infection-related deaths or opportunistic infections
Wesson, 2024 (USA)/retrospective cohort	129/3 months	62/68 (53)	Ciltacabtagene autoleucel, Idecabtagene vicleucel	NR/108 (83.7)	Prophylaxis and G-CSF according to institutional protocols (not standardized)	Incidence, timing, severity, and etiologies of early infections (≤30 days, 15–30 days, >30 days); infection-related NRM	Infections within 30 days in 43/129 (33%), similar for ide-cel and cilta-cel. Most infections occurred during hospitalization or early readmission; pathogens included BK virus, CMV viremia, ESBL-producing bacteria. NRM 2.3%, driven by IEC-HS and sepsis; few late infections and minimal late infectious mortality
Sidana, 2025 (USA)/retrospective cohort	255 (236 evaluable)/13 months	64/145 (57)	Ciltacabtagene autoleucel	Median 6 (range 2–18)/216 (85)	NR	Incidence, type, and grade ≥3 infections; mixed infections; infection-related mortality and contribution to NRM	Infections in 110/236 (47%), with 49 severe (46% of infections). Bacterial infections in 41 (25 severe), viral in 47 (10 severe), fungal in 3 (all severe), mixed in 19 (11 severe). Most infections occurred within 90 days and were associated with prolonged cytopenias. Twelve infection-related deaths (~5%), accounting for 52% of NRM; no excess of unusual pathogens

ANC, absolute neutrophil count; ASCT, autologous stem cell transplantation; AUROC, area under the receiver operating characteristic curve; BCMA, B-cell maturation antigen; BID, twice daily; CAR-T, chimeric antigen receptor T cell; CD4, cluster of differentiation 4; CRS, cytokine release syndrome; CTCAE, Common Terminology Criteria for Adverse Events; ESBL, extended-spectrum beta-lactamase; GI, gastrointestinal; HSCT, hematopoietic stem cell transplantation; ICANS, immune effector cell–associated neurotoxicity syndrome; IgG, immunoglobulin G; IMiD, immunomodulatory drug; IQR, interquartile range; IVIG, intravenous immunoglobulin; NRM, non-relapse mortality; NR, not reported; OR, odds ratio; OS, overall survival; PFS, progression-free survival; PI, proteasome inhibitor; PJP, *Pneumocystis jirovecii* pneumonia; TMP-SMX, trimethoprim–sulfamethoxazole; TID, three times daily; URTI, upper respiratory tract infection.

**Table 2.** Definitions of infection periods and reporting of infectious outcomes across included studies Table 2 long description.

First author, year	N	Early infection period definition (<30 days)	Mild/moderate infections reported	Severe infections reported	Etiology infection reported (bacterial/viral/fungal)	Etiology stratified by time (early vs late)	Infection-related mortality reported
Banerjee, 2021	50			X			
Wang, 2021	40		X	X	X		X
Kambhampati, 2022	55	X	X	X	X	X	X
Logue, 2022	52	X		X	X	X	X
Mi, 2022	48			X			
Wang, 2022	62	X		X	X	X	X
Cheng, 2023	126	X		X	X	X	X
Hammons, 2023	33			X			X
Little, 2023	99			X	X	X	X
Oliver-Caldés, 2023	30			X			X
Rejeski, 2023	113			X			X
Reyes, 2023	83				X		
Zhou, 2023	68	X	X	X	X		X
Caillot, 2024	24	X		X	X	X	X
Dima, 2024	69			X			X
Frenking, 2024	113	X		X			
Trando, 2024	25			X			X
Wesson, 2024	129	X					X
Sidana, 2025	236			X			X

X = outcome reported in the study; Blank cells indicate outcomes that were not reported in the study.

**Figure 2.**
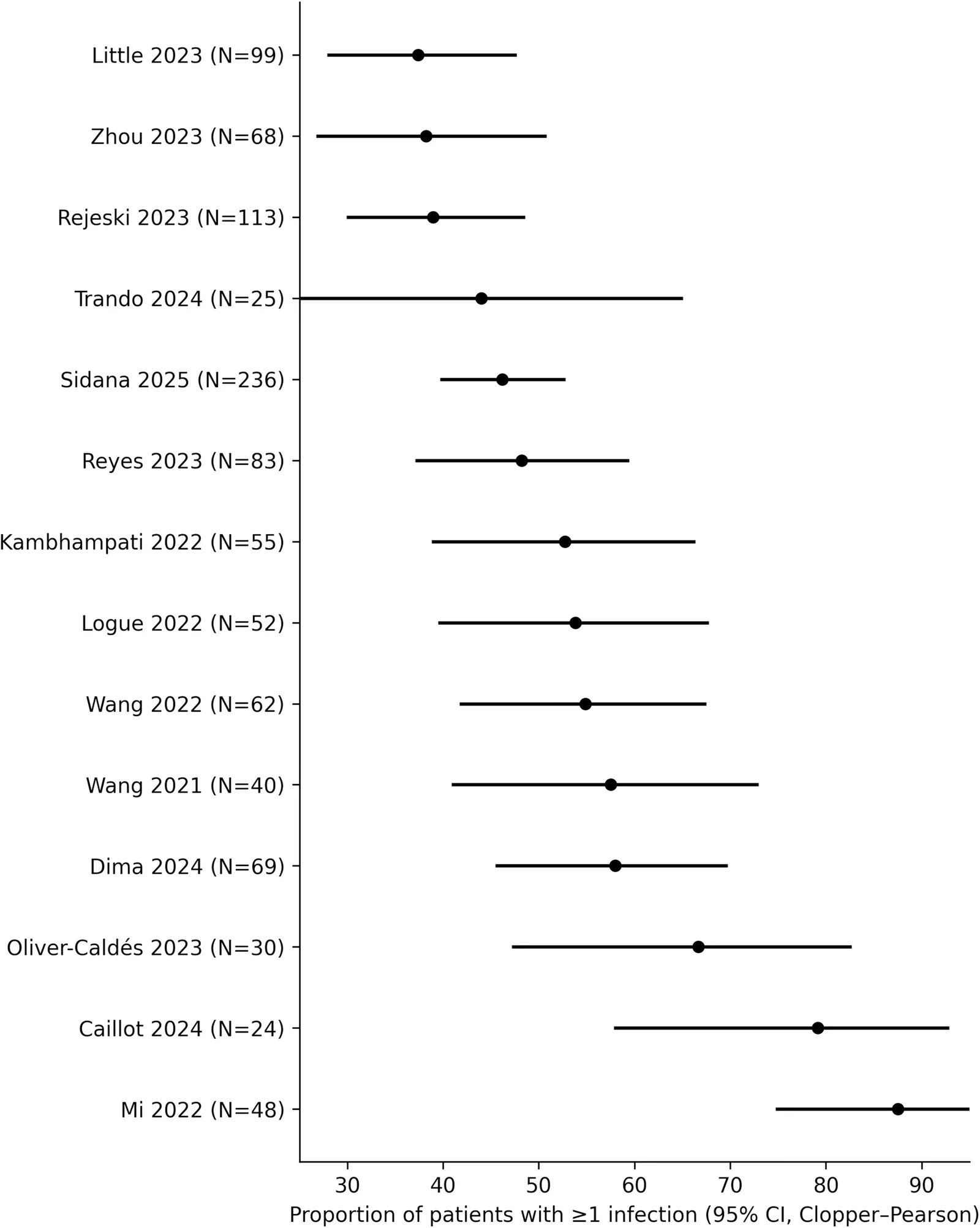
Forest plot of infection incidence across included studies. *Note:* Infection severity definitions were not fully standardized across studies. Although most cohorts used CTCAE criteria, some studies (eg, Zhou 2023 and Rejeski 2023) applied institutional or alternative 5-level clinical grading systems based on treatment intensity and clinical outcomes.

Overall, infectious complications were common following CAR T-cell therapy, with reported incidence ranging from approximately 33% to over 70%. Three cohorts reported infection rates exceeding 70%.^16,23,30^ Five studies were excluded from pooled incidence analysis because patient-level infection data were not reported (Figure 2).^12,19,21,24,25^ Infection incidence varied across idecabtagene vicleucel and ciltacabtagene autoleucel cohorts,^12,14,22,26,28,29^ although rates of severe infection and infection-related mortality overlapped substantially between products.

**Figure 3.**
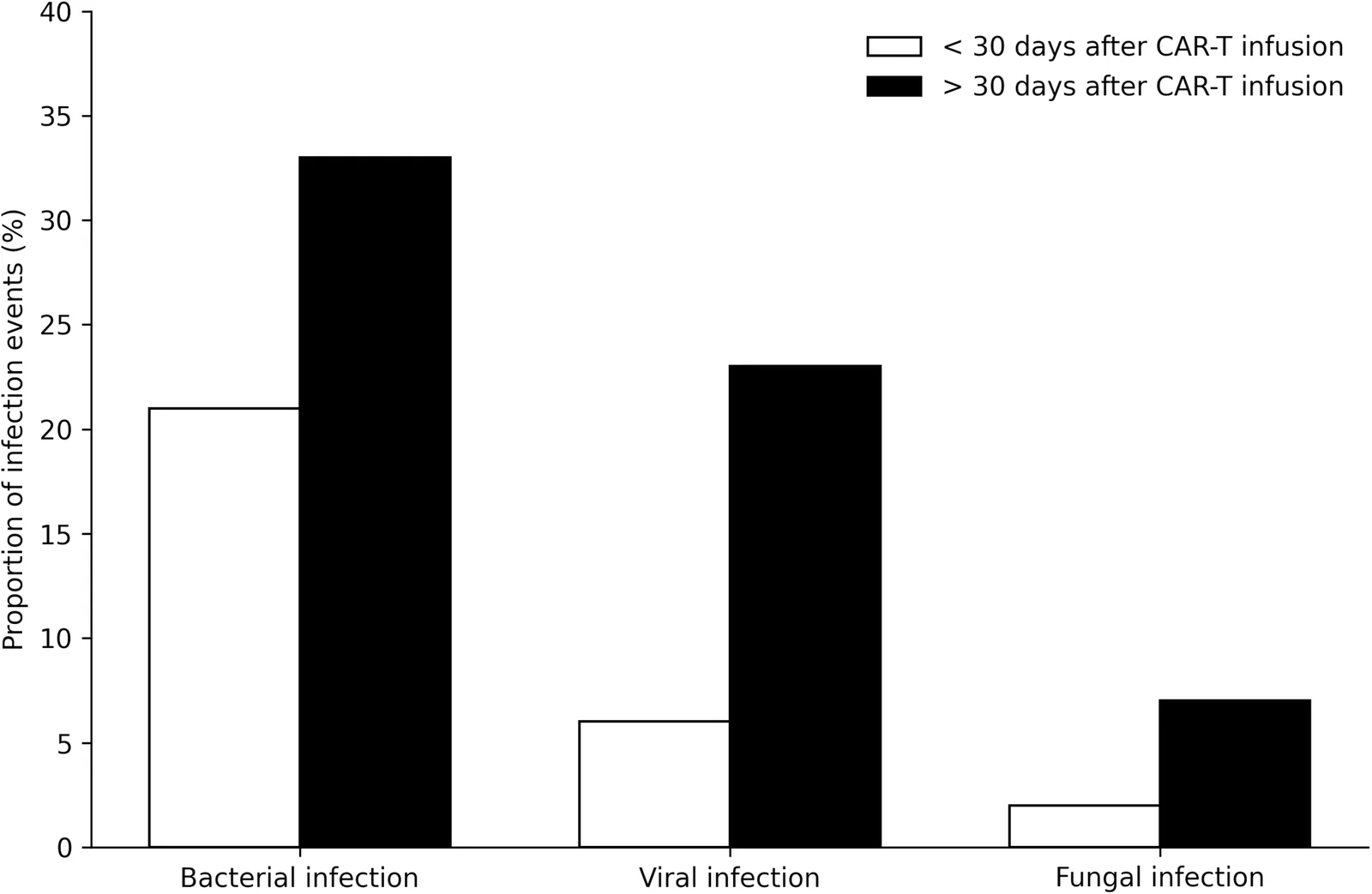
Average proportions of bacterial, viral, and fungal infection events during early and late periods after CAR-T cell infusion. *Note:* The analysis included five studies reporting event-level bacterial, viral, and fungal infections stratified by early (<30 d) and late (≥30 d) periods after CAR T-cell infusion (Caillot, 2024; Cheng, 2023; Logue, 2022; Kambhampati, 2022; Wang, 2022). Proportions were calculated within each study using the total number of reported infectious events as the denominator and summarized as unweighted mean percentages across studies. Studies that did not report data for a given infection category were excluded from the corresponding calculation. Because not all infectious events had a specified etiology, categories do not sum to 100%. Event counts and study-level proportions are shown in Supplementary Tables S1 and S2.

**Figure 4.**
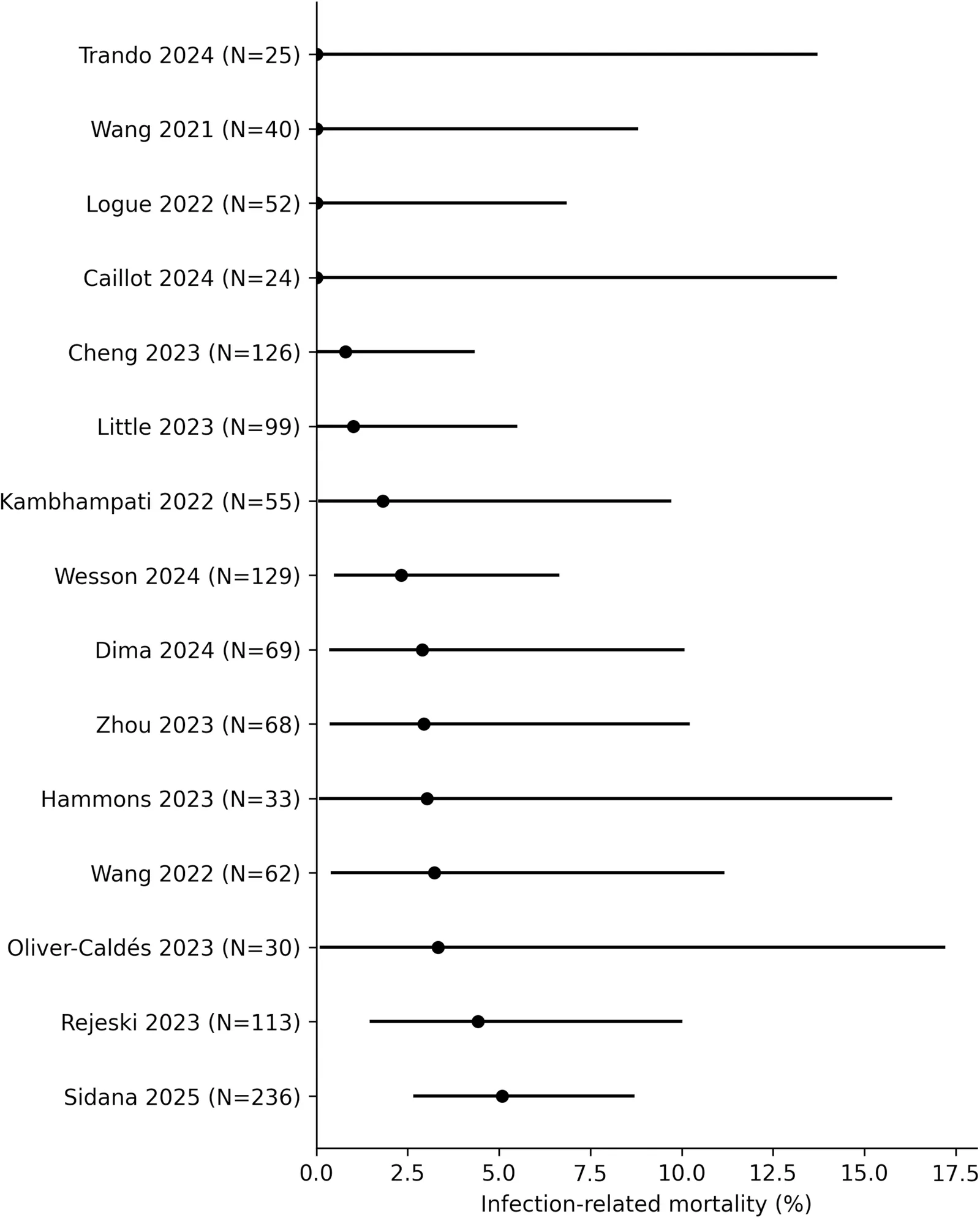
Forest plot of infection-related mortality rates by study.

When reported, bacterial infections accounted for the largest proportion of infectious events, ranging from 19% to 81% across studies, whereas viral infections accounted for 5%–70% and fungal infections generally represented less than 15% of events (5%–12%) (Supplementary Figure S1). Bacterial infections primarily involved the respiratory tract and bloodstream. Viral infections included respiratory viruses, HSV/VZV, and CMV reactivation. Across studies, CMV reactivation was infrequently reported and was generally described as viremia without detailed clinical characterization.^14,23,24,30^ In the cohort reported by Trando et al.,^27^ CMV viremia occurred in 47% of CMV IgG–positive patients within 6 months after ide-cel infusion, with five patients receiving preemptive antiviral therapy initiated at PCR levels ranging from <35 to 1510 IU/mL. Notably, no cases of CMV end-organ disease were observed.^27^ Fungal infections were uncommon and were mainly due to *Candida* species, with occasional invasive mold infections. Across five studies^14,15,19,23,29^ reporting early (<30 d) and late (≥30 d) infections, bacterial infections accounted for a mean of 21% of infectious events during the early period and 33% during the late period, whereas viral infections increased from 6% to 23% of events. Fungal infections remained infrequent, representing only 2% of early and 7% of late infectious events (Figure 3). Study-level analyses demonstrated consistent shifts toward viral predominance during late follow-up, although the magnitude of this increase varied across cohorts (Supplementary Figure S2). For example, viral infections represented 45% of infectious events after day 30 in the study by Kambhampati et al.,^15^ compared with 22% in Logue et al.^14^ and only 4% in Cheng et al.^19^ This analysis was limited to three studies that reported mutually comparable early and late bacterial and viral infection counts.^14,15,19^ Reported rates of severe infection ranged from approximately 5% to nearly 60% across 10 studies^12,14,15,20–22,26–29^ (Supplementary Figure S3). Higher rates of severe infection were consistently observed in cohorts characterized by prolonged cytopenia, elevated CAR-HEMATOTOX scores, impaired immune reconstitution, or prior allogeneic transplantation. Infection-related mortality was reported in 15 studies and varied substantially across cohorts.^13–15,17–24,26,27,29,30^ Observed infection-related mortality rates generally ranged from approximately 0% to over 5%, with wide confidence intervals in smaller cohorts (Figure 4).

Methodological quality assessment using the Downs and Black scale demonstrated predominantly fair (moderate) methodological quality across studies (Table 3). Total scores ranged from 14 to 19 points. Three studies (15.8%) were classified as good quality (score ≥18), whereas the majority, 16 studies (84.2%), were classified as fair quality (scores 14–17).

**Table 3.** Methodological quality assessment of included studies using the Downs and Black scale Table 3 long description.

Study (First author, year)	Reporting (0–11)	External validity (0–3)	Internal validity—bias (0–7)	Internal validity—confounding (0–6)	Power (0–1)	Total (0–28)	Quality
Sidana, 2025	10	1	5	1	0	17	Fair
Wesson, 2024	10	1	5	1	0	17	Fair
Dima, 2024	10	2	5	0	0	17	Fair
Rejeski, 2023	10	0	5	2	0	17	Fair
Little, 2023	9	1	5	1	0	16	Fair
Reyes, 2023	10	2	4	2	0	18	Good
Logue, 2022	10	0	3	1	0	14	Fair
Wang, 2021	10	1	3	2	0	16	Fair
Mi, 2022	10	0	5	3	1	19	Good
Frenking, 2024	9	0	5	2	0	16	Fair
Caillot, 2024	9	0	5	1	0	15	Fair
Hammons, 2024	8	0	5	1	0	14	Fair
Cheng, 2023	10	0	5	2	0	17	Fair
Oliver-Caldés, 2023	9	0	4	1	0	14	Fair
Zhou, 2023	10	0	5	2	0	17	Fair
Kambhampati, 2022	9	0	5	0	0	14	Fair
Banerjee, 2021	9	1	5	2	0	17	Fair
Wang, 2022	10	0	5	2	0	17	Fair
Trando, 2024	11	2	4	2	0	19	Good

*Note:* Downs and Black total score interpretation: good (≥18), fair (14–17), poor (<14). Question 27 was adapted to a binary format (yes/no), resulting in a maximum total score of 28. Quality distribution: Good = 3 studies (15.8%), Fair = 16 studies (84.2%).

## Discussion

In this systematic review, we synthesized data from 19 studies encompassing 1,495 patients with relapsed or refractory multiple myeloma treated with CAR T-cell therapy, the majority of whom were heavily pretreated and had undergone prior autologous stem cell transplantation. Across cohorts, infectious complications were frequent, with 33%–70% of patients experiencing at least one infectious case. Recurrent risk factors included prolonged neutropenia and other persistent cytopenia, low CD4^+^ T-cell counts, hypogammaglobulinemia, CRS, and suboptimal disease response. Severe infections ranged from approximately 5% to nearly 60% across cohorts,^12,14,15,20–22,26–29^ and infection-related mortality ranged from approximately 0% to 5%.

Our findings expand upon prior reviews addressing infections after CAR T-cell therapy. Lombardi et al.^6^ reported infection rates ranging from 26% to 39% based largely on registration studies of platforms, including CAR T cells, but with limited follow-up, and heterogeneous underlying diseases. Similarly, the systematic review and meta-analysis by Telli Dizman et al.^7^ provided pooled infection estimates across hematologic malignancies; however, the estimate for multiple myeloma (approximately 35%) was derived from only six studies comprising 308 patients and was characterized by wide confidence intervals and heterogeneity. By contrast, our review deliberately focused on multiple myeloma and included a larger and more contemporary patient population, allowing a more granular assessment of infection in real-world and postapproval settings.

In our review, infections predominantly occurred within the first 30 days after infusion. During this early phase, bacterial infections predominated, whereas viral infections accounted for approximately 6% of events.^31^ Beyond 30 days, we observed a marked shift in the infectious profile, with viral infections increasing to approximately 23% of reported events, while bacterial infections remained the most prevalent. This temporal pattern is similar to that observed after hematopoietic cell transplantation (HCT) and solid organ transplantation, where early bacterial infections are followed by an increasing burden of viral infections, supporting a time-dependent approach to infection surveillance and prophylaxis after anti-BCMA CAR-T cell therapy.^32,33^

Bacterial infections most commonly involved the respiratory tract and bloodstream.^14,17,24,30^ Prior reviews similarly identified bacterial infections as the most frequent etiology after CAR T-cell therapy but did not provide pathogen-level detail.^6,7^ Most cohorts reported infections at the level of clinical syndrome or pathogen class rather than providing systematic microbiological characterization. Only a few studies described specific pathogens associated with infectious events, including bacterial bloodstream infections, respiratory viral pathogens, or invasive fungal infections.^18,19,24,26,30^ Furthermore, only 4 of the 19 included studies required microbiological confirmation of infectious events, highlighting the heterogeneity in infection definitions across the available literature.^13,17,19,28^ Viral infections included respiratory viruses, HSV/VZV, and CMV.^14,18,19,24,26,30^ Respiratory viral infections were generally reported as aggregated categories such as “upper respiratory tract infection” or “respiratory viral infection,” although a few studies specified pathogens including rhinovirus, parainfluenza virus, seasonal coronaviruses, and SARS-CoV-2.^14,24,30^ Fungal infections accounted for a smaller fraction of events, generally below 10%–15%, and were predominantly caused by *Candida* species, with occasional invasive mold infections, particularly *Aspergillus* species. Although fungal infections represented a higher relative proportion after 30 days (7% of events) compared with the early period (2% of events) (Figure 3), the absolute number of fungal infections remained low overall, with only 4 early (<30 d) and 14 late (≥30 d). This finding should therefore be interpreted cautiously given the limited number of studies contributing comparable early and late infection data.^15,19,23,29^ The observed pattern may reflect persistent cytopenia, corticosteroid exposure, or delayed immune reconstitution following CAR-T therapy.^1,34,35^

Supportive care and anti-infective prophylaxis strategies were reported in most studies but varied considerably. Antiviral prophylaxis with acyclovir or valacyclovir and prophylaxis against *Pneumocystis jirovecii* pneumonia were routinely implemented in most cohorts, consistent with expert recommendations extrapolated from hematopoietic stem cell transplantation practices.^31,34^ Notably, no confirmed cases of pneumocystosis were reported in our review, suggesting a potential protective effect. In contrast, antibacterial and antifungal prophylaxis were more heterogeneous and generally restricted to periods of prolonged neutropenia or high-dose corticosteroid exposure.^2,35^

In our review, severe infection rates ranged from approximately 5% to nearly 60% across cohorts (Supplementary Figure S3). Severe infections were associated with hospitalization, intensive care unit admission, and intravenous antimicrobial therapy, particularly during the early period following CAR T-cell therapy. Infection-related mortality ranged from approximately 0% to over 5% (Figure 4), consistent with broader evidence that infections remain an important contributor to non-relapse morbidity and mortality following CAR T-cell therapy.^1,5,7^ However, assessment of the true clinical impact remains limited, as key end points such as infection-free survival and infection-related non-relapse mortality were evaluated in only three studies. These findings reinforce the importance of structured clinical monitoring and longitudinal infectious surveillance, particularly for patients at higher risk complications. Future studies should aim to characterize infection patterns according to the type and degree of immune dysfunction following CAR-T therapy, such as prolonged CD4^+^ T-cell lymphopenia versus isolated B-cell aplasia. Such stratification could help clarify pathogen-specific risks (eg, CMV reactivation or opportunistic infections) and support more individualized surveillance and prophylaxis strategies.

This study has several limitations. Most included studies were retrospective and heterogeneous in design, follow-up duration, and infection definitions, limiting direct comparability across cohorts. Reporting of infection severity, etiologic classification, antimicrobial resistance patterns, and immune reconstitution parameters was inconsistent, precluding more granular pooled analyses. Temporal stratification beyond the <30 and ≥30-day framework was not feasible due to variability in reporting. In addition, attribution of mortality to infectious causes varied between studies, and underdiagnosis of certain infections, particularly viral reactivations and opportunistic pathogens, cannot be excluded. Another limitation of the available literature is the lack of information regarding vaccination status and immunization strategies. None of the included studies systematically reported baseline vaccination history or revaccination schedules following CAR-T cell therapy, limiting the ability to assess the potential impact of immunization on infection risk. Finally, as CAR T-cell therapy remains a relatively recent intervention, data on long-term immune recovery and late infectious complications remain limited.

In conclusion, despite its efficacy, CAR T-cell therapy in multiple myeloma is associated with a substantial burden of infectious complications, characterized by high overall incidence, predominance of bacterial infections in the early period. Standardized infection definitions, harmonized follow-up, and integrated immune monitoring are critical to improve risk stratification, optimize prevention strategies, and reduce infectious morbidity and mortality, while preserving the transformative benefits of CAR T-cell therapy in multiple myeloma.

## Supporting information

10.1017/ash.2026.10830.sm001 Cotia et al. supplementary material Cotia et al. supplementary material

## Data Availability

This study generated no new data sets. All data analyzed were obtained from published articles and are available within the manuscript and supplementary files.

## Supplementary material

The supplementary material for this article can be found at https://doi.org/10.1017/ash.2026.10830.

## Table 1. Long description

A table with 30 rows and 10 columns summarizing studies on infectious complications after CAR T-cell therapy in multiple myeloma. The columns are Author, Year, Study Design, Number of Patients, CAR T-cell Product, Primary Endpoint, Infectious Complications, Predictors of Infections, Supportive Care, and Prophylactic Strategies. Row 1: Author, Kite; Year, 2017; Study Design, Single-arm; Number of Patients, 79; CAR T-cell Product, axi-cel; Primary Endpoint, Overall Response Rate; Infectious Complications, 33 percent; Predictors of Infections, CRS, ICANS; Supportive Care, IVIG, G-CSF; Prophylactic Strategies, Acyclovir, Pneumocystis jirovecii pneumonia prophylaxis. Row 2: Author, JNJ; Year, 2018; Study Design, Single-arm; Number of Patients, 33; CAR T-cell Product, JNJ-4528; Primary Endpoint, Overall Response Rate; Infectious Complications, 42 percent; Predictors of Infections, CRS, ICANS; Supportive Care, IVIG, G-CSF; Prophylactic Strategies, Acyclovir, Pneumocystis jirovecii pneumonia prophylaxis. Row 3: Author, Bluebird Bio; Year, 2018; Study Design, Single-arm; Number of Patients, 43; CAR T-cell Product, bb2121; Primary Endpoint, Overall Response Rate; Infectious Complications, 28 percent; Predictors of Infections, CRS, ICANS; Supportive Care, IVIG, G-CSF; Prophylactic Strategies, Acyclovir, Pneumocystis jirovecii pneumonia prophylaxis. Row 4: Author, Legend; Year, 2019; Study Design, Single-arm; Number of Patients, 74; CAR T-cell Product, liso-cel; Primary Endpoint, Overall Response Rate; Infectious Complications, 35 percent; Predictors of Infections, CRS, ICANS; Supportive Care, IVIG, G-CSF; Prophylactic Strategies, Acyclovir, Pneumocystis jirovecii pneumonia prophylaxis. Row 5: Author, Bristol Myers Squibb; Year, 2019; Study Design, Single-arm; Number of Patients, 41; CAR T-cell Product, ide-cel; Primary Endpoint, Overall Response Rate; Infectious Complications, 39 percent; Predictors of Infections, CRS, ICANS; Supportive Care, IVIG, G-CSF; Prophylactic Strategies, Acyclovir, Pneumocystis jirovecii pneumonia prophylaxis. Row 6: Author, Pinnacle; Year, 2020; Study Design, Single-arm; Number of Patients, 62; CAR T-cell Product, P-BCMA-101; Primary Endpoint, Overall Response Rate; Infectious Complications, 30 percent; Predictors of Infections, CRS, ICANS; Supportive Care, IVIG, G-CSF; Prophylactic Strategies, Acyclovir, Pneumocystis jirovecii pneumonia prophylaxis. Row 7: Author, Celgene; Year, 2020; Study Design, Single-arm; Number of Patients, 29; CAR T-cell Product, LCAR-B38M; Primary Endpoint, Overall Response Rate; Infectious Complications, 31 percent; Predictors of Infections, CRS, ICANS; Supportive Care, IVIG, G-CSF; Prophylactic Strategies, Acyclovir, Pneumocystis jirovecii pneumonia prophylaxis. Row 8: Author, Fujian; Year, 2020; Study Design, Single-arm; Number of Patients, 17; CAR T-cell Product, FT819; Primary Endpoint, Overall Response Rate; Infectious Complications, 29 percent; Predictors of Infections, CRS, ICANS; Supportive Care, IVIG, G-CSF; Prophylactic Strategies, Acyclovir, Pneumocystis jirovecii pneumonia prophylaxis. Row 9: Author, Shanghai; Year, 2020; Study Design, Single-arm; Number of Patients, 19; CAR T-cell Product, RECAR20; Primary Endpoint, Overall Response Rate; Infectious Complications, 33 percent; Predictors of Infections, CRS, ICANS; Supportive Care, IVIG, G-CSF; Prophylactic Strategies, Acyclovir, Pneumocystis jirovecii pneumonia prophylaxis. Row 10: Author, Jiangsu; Year, 2020; Study Design, Single-arm; Number of Patients, 20; CAR T-cell Product, CT053; Primary Endpoint, Overall Response Rate; Infectious Complications, 35 percent; Predictors of Infections, CRS, ICANS; Supportive Care, IVIG, G-CSF; Prophylactic Strategies, Acyclovir, Pneumocystis jirovecii pneumonia prophylaxis.

Navigate back to Table 1..

## Table 2. Long description

A table comparing the reporting of infectious outcomes across various studies, detailing infection periods, severity, etiology, and mortality. The table has 17 rows and 6 columns. The columns are labeled as follows: First author, year; N; Early infection period definition (< 30 days); Mild/moderate infections reported; Severe infections reported; Etiology infection reported (bacterial/viral/fungal); Etiology stratified by time (early vs late); Infection-related mortality reported. The rows are labeled with the first author’s name, year of publication, and various study details. Row 1: Banerjee, 2021; 50; X; X; X; X; X. Row 2: Wang, 2021; 40; X; X; X; X; X. Row 3: Kambhampati, 2022; 55; X; X; X; X; X. Row 4: Logue, 2022; 52; X; X; X; X; X. Row 5: Mi, 2022; 48; X; X; X; X; X. Row 6: Wang, 2022; 62; X; X; X; X; X. Row 7: Cheng, 2023; 126; X; X; X; X; X. Row 8: Hammons, 2023; 33; X; X; X; X; X. Row 9: Little, 2023; 99; X; X; X; X; X. Row 10: Oliver-Caldés, 2023; 30; X; X; X; X; X. Row 11: Rejeski, 2023; 113; X; X; X; X; X. Row 12: Reyes, 2023; 83; X; X; X; X; X. Row 13: Zhou, 2023; 68; X; X; X; X; X. Row 14: Caillot, 2024; 24; X; X; X; X; X. Row 15: Dima, 2024; 69; X; X; X; X; X. Row 16: Frenking, 2024; 113; X; X; X; X; X. Row 17: Trando, 2024; 25; X; X; X; X; X. Row 18: Wesson, 2024; 129; X; X; X; X; X. Row 19: Sidana, 2025; 236; X; X; X; X; X.

Navigate back to Table 2..

## Table 3. Long description

A table comparing the methodological quality of studies using the Downs and Black scale. The table has 19 rows and 6 columns. The columns are labeled Study (First author, year), Reporting (0-11), External validity (0-3), Internal validity—bias (0-7), Internal validity—confounding (0-6), Power (0-1), and Total Quality (0-28). Each row lists a study with corresponding scores in each category. Notable studies include Sidana, 2025; Wesson, 2024; Dima, 2024; Rejeski, 2023; Little, 2023; Reyes, 2023; Logue, 2022; Wang, 2021; Mi, 2022; Frenking, 2024; Caillot, 2024; Hammons, 2024; Cheng, 2023; Oliver-Caldés, 2023; Zhou, 2023; Kambhampati, 2022; Banerjee, 2021; Wang, 2022; Trando, 2024. The total quality scores range from 14 to 19 points, with most studies classified as fair quality.

Navigate back to Table 3..
