# Supplementary material for: Infectious complications after anti-BCMA CAR-T cell therapy in patients with multiple myeloma: a systematic literature review
Source: Antimicrob Steward Healthc Epidemiol. 2026 Sep 9;6(1):e263. doi: 10.1017/ash.2026.10830 (PMC13760433; doi:10.1017/ash.2026.10830)
Supplement: Supplementary file 1 [file S2732494X26108304sup001.docx]

**Infectious Complications After CAR-T Cell Therapy in Patients with Multiple Myeloma: A Systematic Literature Review**

Supplemental Material

Arthur Franco Cotia^1^, MD; André Luís Franco Cotia^2^, MD; Beatriz Bonini Zancopé^3^; Newton de Freitas Centurião^2^, MD; Mariana Kim Hsieh^4^, MD; Erika Viana-Cardenas^5^, MD; Patricia Deffune Celeghini^2^, MD; Pietra Sovero^3^; Isabele Pardo^3^; Vinicius S. Marra^7^; Carlos Henrique Spadão Tudino^3^; Jorge L. Salinas^5^, MD; Alex N. Zimmet^5^, MD; Pedro Hsieh^7^, MD; Nelson Hamerschlak^2^, MD; Mariana Nassif Kerbauy^2^, MD; Michael B. Edmond^6^ MD; Alexandre R. Marra^2,7^, MD

1. Universidade Federal de São Paulo (UNIFESP), São Paulo SP, Brazil
2. Hospital Israelita Albert Einstein, São Paulo, SP, Brazil
3. Faculdade Israelita de Ciências da Saúde Albert Einstein, Hospital Israelita Albert Einstein, São Paulo, SP, Brazil
4. Program of Hospital Epidemiology, University of Iowa Health Care, Iowa City, IA, USA
5. Division of Infectious Diseases & Geographic Medicine, Stanford University School of Medicine, Stanford, CA, USA
6. Department of Medicine, West Virginia University School of Medicine, Morgantown, WV, USA
7. University of Iowa, Iowa City, IA, USA

1. Contents

1. **Cover Page** …………………………………………………….…………………..……………………. 1
2. **Contents** ……………………………………….……….……………………….……………….......... 2
3. **Supplementary Figure S1**. …..…………………………………….………..…………………... 3
4. **Supplementary Figure S2.** ……...................................................................... 4
5. **Supplementary Figure S3.** ………………………………..…………….…………..………..…. 5
6. **Supplementary Table S1.** ..….……………………………..…………….………………..……. 6
7. **Supplementary Table S2.** ..….……………………………..…………….………………..……. 7
8. **Search Strategy** ….……………………………..…………….………………..………………….…. 8

Supplementary Figure S1. Etiological involvement of infection events across CAR-T cell therapy studies

Supplementary Figure S2. Early and late bacterial and viral infection involvement following CAR-T cell therapy

Supplementary Figure S3. Severe infection rates across the included studies

Supplementary Table S1. Event-level infectious counts by timing and etiology

| **Study** | **Total infectious events** | **Early bacterial** | **Early viral** | **Early fungal** | **Late bacterial** | **Late viral** | **Late fungal** |
| --- | --- | --- | --- | --- | --- | --- | --- |
| Caillot, 2024 | 27 | 5 | NR | 1 | 0 | NR | 2 |
| Cheng, 2023 | 85 | 21 | 1 | 3 | 45 | 3 | 6 |
| Logue, 2022 | 46 | 15 | 4 | NR | 12 | 10 | NR |
| Kambhampati, 2022 | 47 | 3 | 4 | 0 | 16 | 21 | 3 |
| Wang, 2022 | 34 | NR | NR | NR | 18 | 8 | 3 |

NR: not reported.

Supplementary Table S2. Study-level proportions of infectious events by timing and etiology

| **Study** | **Total infectious events** | **Early bacterial** | **Early viral** | **Early fungal** | **Late bacterial** | **Late viral** | **Late fungal** |
| --- | --- | --- | --- | --- | --- | --- | --- |
| Caillot, 2024 | 27 | 19% | NR | 4% | 0% | NR | 7% |
| Cheng, 2023 | 85 | 25% | 1% | 4% | 53% | 4% | 7% |
| Logue, 2022 | 46 | 33% | 9% | NR | 26% | 22% | NR |
| Kambhampati, 2022 | 47 | 6% | 9% | 0% | 34% | 45% | 6% |
| Wang, 2022 | 34 | NR | NR | NR | 53% | 24% | 9% |
| Mean |  | 21% | 6% | 2% | 33% | 23% | 7% |

NR: not reported. Mean values were calculated across studies excluding missing values (NR) for each infection category.

Search Strategy

((((((((((((((((((((((((((((((((("hematologic neoplasms"[MeSH Terms]) OR ("bone marrow neoplasms"[MeSH Terms])) OR ("bone marrow failure disorders"[MeSH Terms])) OR ("leukemia"[MeSH Terms])) OR ("leukemia, lymphoid"[MeSH Terms])) OR ("leukemia, myeloid"[MeSH Terms])) OR ("leukemia, myeloid, acute"[MeSH Terms])) OR ("precursor cell lymphoblastic leukemia lymphoma"[MeSH Terms])) OR ("leukemia, myelogenous, chronic, bcr abl positive"[MeSH Terms])) OR ("leukemia, lymphocytic, chronic, b cell"[MeSH Terms])) OR ("leukemia, t cell"[MeSH Terms])) OR ("leukemia, biphenotypic, acute"[MeSH Terms])) OR ("lymphoma"[MeSH Terms])) OR ("lymphoma, non hodgkin"[MeSH Terms])) OR ("hodgkin disease"[MeSH Terms])) OR ("lymphoma, t cell"[MeSH Terms])) OR ("lymphoma, b cell"[MeSH Terms])) OR ("lymphoma, b cell, marginal zone"[MeSH Terms])) OR ("burkitt lymphoma"[MeSH Terms])) OR ("lymphoma, t cell, peripheral"[MeSH Terms])) OR ("lymphoma, large cell, anaplastic"[MeSH Terms])) OR ("lymphoma, t cell, cutaneous"[MeSH Terms])) OR ("lymphoma, large b cell, diffuse"[MeSH Terms])) OR ("lymphoma, mantle cell"[MeSH Terms])) OR ("multiple myeloma"[MeSH Terms])) OR ("leukemia, plasma cell"[MeSH Terms])) OR ("monoclonal gammopathy of undetermined significance"[MeSH Terms])) OR ("smoldering multiple myeloma"[MeSH Terms])) OR ("immunoglobulin light chain amyloidosis"[MeSH Terms])) OR ("lymphoma, follicular"[MeSH Terms])) AND (((("immunotherapy, adoptive"[MeSH Terms]) OR ("receptors, chimeric antigen"[MeSH Terms])) OR ("b cell maturation antigen"[MeSH Terms])) OR ("antigens, cd19"[MeSH Terms]))) AND (((((((((((((((((((((((((((((((((((("drug related side effects and adverse reactions"[MeSH Terms]) OR ("infections"[MeSH Terms])) OR ("febrile neutropenia"[MeSH Terms])) OR ("cytomegalovirus infections"[MeSH Terms])) OR ("virus diseases"[MeSH Terms])) OR ("bacterial infections"[MeSH Terms])) OR ("fungi"[MeSH Terms])) OR ("tuberculosis"[MeSH Terms])) OR ("parasitic diseases"[MeSH Terms])) OR ("sepsis"[MeSH Terms])) OR ("abscess"[MeSH Terms])) OR ("covid 19"[MeSH Terms])) OR ("mycoses"[MeSH Terms])) OR ("encephalomyelitis"[MeSH Terms])) OR ("encephalitis"[MeSH Terms])) OR ("meningoencephalitis"[MeSH Terms])) OR ("myelitis"[MeSH Terms])) OR ("opportunistic infections"[MeSH Terms])) OR ("respiratory tract infections"[MeSH Terms])) OR ("pneumonia"[MeSH Terms])) OR ("tracheitis"[MeSH Terms])) OR ("bacteremia"[MeSH Terms])) OR ("fungemia"[MeSH Terms])) OR ("skin diseases"[MeSH Terms])) OR ("soft tissue infections"[MeSH Terms])) OR ("suppuration"[MeSH Terms])) OR ("urinary tract infections"[MeSH Terms])) OR ("hepatitis"[MeSH Terms])) OR ("viremia"[MeSH Terms])) OR ("herpesvirus 1, human"[MeSH Terms])) OR ("herpesvirus 2, human"[MeSH Terms])) OR ("herpesvirus 3, human"[MeSH Terms])) OR ("varicella zoster virus infection"[MeSH Terms])) OR ("epstein barr virus infections"[MeSH Terms])) OR ("virus diseases"[MeSH Terms])) OR ("herpesviridae infections"[MeSH Terms]))) OR ((((((((((((((((((((((((((((((((((((((((((((((((((((((((((((((((((((((((((("hematologic neoplasms"[Title/Abstract]) OR ("hematologic neoplasm"[Title/Abstract])) OR ("bone marrow neoplasms"[Title/Abstract])) OR ("bone marrow neoplasm"[Title/Abstract])) OR ("bone marrow failure disorders"[Title/Abstract])) OR ("bone marrow failure disorder"[Title/Abstract])) OR ("leukemia"[Title/Abstract])) OR ("lymphoid leukemia"[Title/Abstract])) OR ("myeloid leukemia"[Title/Abstract])) OR ("acute myeloid leukemia"[Title/Abstract]))) OR ("precursor cell lymphoblastic leukemia lymphoma"[Title/Abstract])) OR ("bcr abl chronic myelogenous leukemia"[Title/Abstract])) OR ("b cell chronic lymphocytic leukemia"[Title/Abstract])) OR ("t cell leukemia"[Title/Abstract])) OR ("acute biphenotypic leukemia"[Title/Abstract])) OR ("lymphoma"[Title/Abstract])) OR ("lymphomas"[Title/Abstract])) OR ("non hodgkin lymphoma"[Title/Abstract])) OR ("hodgkin disease"[Title/Abstract])) OR ("t cell lymphoma"[Title/Abstract])) OR ("b cell lymphoma"[Title/Abstract])) OR ("marginal zone b cell lymphoma"[Title/Abstract])) OR ("burkitt lymphoma"[Title/Abstract])) OR ("peripheral t cell lymphoma"[Title/Abstract])) OR ("anaplastic large cell lymphoma"[Title/Abstract])) OR ("cutaneous t cell lymphoma"[Title/Abstract])) OR ("diffuse large b cell lymphoma"[Title/Abstract])) OR ("mantle cell lymphoma"[Title/Abstract])) OR ("multiple myeloma"[Title/Abstract])) OR ("plasma cell leukemia"[Title/Abstract])) OR ("monoclonal gammopathy of undetermined significance"[Title/Abstract])) OR ("smoldering multiple myeloma"[Title/Abstract])) OR ("immunoglobulin light chain amyloidosis"[Title/Abstract])) OR ("hematologic malignancies"[Title/Abstract])) OR ("hematologic tumors"[Title/Abstract])) OR ("hematological cancer"[Title/Abstract])) OR ("hematological pathologies"[Title/Abstract] OR "hematological pathology"[Title/Abstract])) OR ("blood neoplasm"[Title/Abstract])) OR ("blood neoplasms"[Title/Abstract])) OR ("blood malignancies"[Title/Abstract])) OR ("blood malignancy"[Title/Abstract])) OR ("hemopoietic neoplasm"[Title/Abstract])) OR ("hemopoietic neoplasms"[Title/Abstract] OR "hemopoietic neoplastic"[Title/Abstract])) OR ("bone marrow failure syndrome"[Title/Abstract])) OR ("bone marrow failure syndromes"[Title/Abstract])) OR ("acute leukemia"[Title/Abstract])) OR ("chronic leukemia"[Title/Abstract])) OR ("acute myeloid leukemia"[Title/Abstract])) OR ("acute lymphocytic leukemia"[Title/Abstract])) OR ("chronic myelogenous leukemia"[Title/Abstract])) OR ("chronic myelogenous leukemias"[Title/Abstract])) OR ("chronic lymphocytic leukemia"[Title/Abstract])) OR ("b cell leukemia"[Title/Abstract])) OR ("b cell acute lymphocytic leukemia"[Title/Abstract])) OR ("t cell acute lymphoblastic leukemia"[Title/Abstract])) OR ("t cell chronic lymphocytic leukemia"[Title/Abstract])) OR ("hodgkin lymphoma"[Title/Abstract])) OR ("marginal zone lymphoma"[Title/Abstract])) OR ("nodular lymphocyte predominant hodgkin lymphoma"[Title/Abstract])) OR ("extranodal lymphoma"[Title/Abstract])) OR ("lymphoblastic lymphoma t cell"[Title/Abstract])) OR ("t cell large cell lymphoma"[Title/Abstract])) OR ("t cell peripheral lymphoma"[Title/Abstract])) OR ("t cell extranodal lymphomas"[Title/Abstract])) OR ("t cell extranodal lymphomas"[Title/Abstract])) OR ("plasma cell myeloma"[Title/Abstract])) OR ("light chain amyloidosis"[Title/Abstract])) OR ("iga myeloma"[Title/Abstract])) OR ("igg myeloma"[Title/Abstract])) OR ("nonsecretory multiple myeloma"[Title/Abstract])) OR ("follicular lymphoma"[Title/Abstract])) OR ("b cell precursor acute lymphoblastic leukemia"[Title/Abstract])) AND ((((((((((((((((((((((((((((((((((((("adoptive immunotherapy"[Title/Abstract]) OR ("adoptive immunotherapies"[Title/Abstract])) OR ("chimeric antigen receptors"[Title/Abstract])) OR ("chimeric antigen receptor t cell"[Title/Abstract])) OR ("chimeric t cell receptor"[Title/Abstract])) OR ("chimeric t cell receptors"[Title/Abstract])) OR ("chimeric immunoreceptor"[Title/Abstract] OR "chimeric immunoreceptors"[Title/Abstract])) OR ("chimeric antigen receptor therapy"[Title/Abstract])) OR ("chimeric antigen receptor therapies"[Title/Abstract])) OR ("tisagenlecleucel"[Title/Abstract])) OR ("kymriah"[Title/Abstract])) OR ("axicabtagene ciloleucel"[Title/Abstract])) OR ("yescarta"[Title/Abstract])) OR ("brexucabtagene autoleucel"[Title/Abstract])) OR ("tecartus"[Title/Abstract])) OR ("idecabtagene vicleucel"[Title/Abstract])) OR ("abecma"[Title/Abstract])) OR ("lisocabtagene maraleucel"[Title/Abstract])) OR ("breyanzi"[Title/Abstract])) OR ("ciltacabtagene"[Title/Abstract])) OR ("ciltacabtagene autoleucel"[Title/Abstract])) OR ("carvykti"[Title/Abstract])) OR ("cd19 chimeric antigen receptor"[Title/Abstract])) OR ("cd19 chimeric antigen receptors"[Title/Abstract])) OR ("car t cell"[Title/Abstract])) OR ("car t cells"[Title/Abstract])) OR ("adoptive cellular immunotherapy"[Title/Abstract])) OR ("adoptive cellular immunotherapies"[Title/Abstract])) OR ("adoptive immunotherapy"[Title/Abstract])) OR ("adoptive immunotherapies"[Title/Abstract])) OR ("car t cell therapy"[Title/Abstract])) OR ("car t cell therapies"[Title/Abstract])) OR ("chimeric antigen receptor"[Title/Abstract])) OR ("chimeric antigen receptors"[Title/Abstract])) OR ("artificial t cell receptor"[Title/Abstract] OR "artificial t cell receptors"[Title/Abstract])) OR ("b cell maturation antigen"[Title/Abstract])) OR ("cd19 antigens"[Title/Abstract]))) AND ((((((((((((((((((((((((((((((((((((((((((((((((((((((((((((((((((((((((((((((((((((((((((("drug related side effects and adverse reactions"[Title/Abstract]) OR ("infections"[Title/Abstract])) OR ("infection"[Title/Abstract])) OR ("febrile neutropenia"[Title/Abstract])) OR ("cytomegalovirus infections"[Title/Abstract])) OR ("cytomegalovirus infection"[Title/Abstract])) OR ("virus diseases"[Title/Abstract])) OR (Virus Diseases[Title/Abstract])) OR ("virus disease"[Title/Abstract])) OR ("bacterial infection"[Title/Abstract])) OR ("bacterial infections"[Title/Abstract])) OR ("fungi"[Title/Abstract])) OR ("tuberculosis"[Title/Abstract])) OR ("parasitic disease"[Title/Abstract])) OR ("parasitic diseases"[Title/Abstract])) OR ("sepsis"[Title/Abstract])) OR ("abscess"[Title/Abstract])) OR ("covid 19"[Title/Abstract])) OR ("mycoses"[Title/Abstract])) OR ("encephalomyelitis"[Title/Abstract])) OR ("encephalitis"[Title/Abstract])) OR ("meningoencephalitis"[Title/Abstract])) OR ("myelitis"[Title/Abstract])) OR ("opportunistic infections"[Title/Abstract])) OR ("opportunistic infection"[Title/Abstract])) OR ("respiratory tract infections"[Title/Abstract])) OR ("respiratory tract infection"[Title/Abstract])) OR ("pneumonia"[Title/Abstract])) OR ("tracheitis"[Title/Abstract])) OR ("bacteremia"[Title/Abstract])) OR ("fungemia"[Title/Abstract])) OR ("skin diseases"[Title/Abstract])) OR ("skin disease"[Title/Abstract])) OR ("soft tissue infections"[Title/Abstract])) OR ("soft tissue infection"[Title/Abstract])) OR ("suppuration"[Title/Abstract])) OR ("urinary tract infections"[Title/Abstract])) OR ("urinary tract infection"[Title/Abstract])) OR ("hepatitis"[Title/Abstract])) OR ("viremia"[Title/Abstract])) OR ("human herpesvirus 1"[Title/Abstract] OR "human herpesvirus 1 infection"[Title/Abstract] OR "human herpesvirus 2"[Title/Abstract] OR "human herpesvirus 3"[Title/Abstract])) OR ("varicella zoster virus infection"[Title/Abstract])) OR ("varicella zoster virus infections"[Title/Abstract])) OR ("epstein barr virus infection"[Title/Abstract])) OR ("epstein barr virus infections"[Title/Abstract])) OR ("virus disease"[Title/Abstract])) OR ("virus diseases"[Title/Abstract])) OR ("herpesviridae infections"[Title/Abstract])) OR ("herpesviridae infection"[Title/Abstract])) OR ("drug related side effects"[Title/Abstract])) OR ("drug related side effect"[Title/Abstract])) OR ("adverse reactions included"[Title/Abstract])) OR ("adverse drug reactions"[Title/Abstract])) OR ("adverse drug reaction"[Title/Abstract])) OR ("drug reactions"[Title/Abstract])) OR ("adverse drug reaction"[Title/Abstract])) OR ("drug toxicity"[Title/Abstract])) OR ("drug toxicities"[Title/Abstract])) OR ("adverse events"[Title/Abstract])) OR ("adverse event"[Title/Abstract])) OR ("pulmonary fungal diseases"[Title/Abstract])) OR ("pulmonary fungal disease"[Title/Abstract])) OR ("fungal infection"[Title/Abstract])) OR ("fungal infections"[Title/Abstract])) OR ("fungi infection"[Title/Abstract])) OR ("fungi infections"[Title/Abstract])) OR ("virus infection"[Title/Abstract])) OR ("virus infections"[Title/Abstract])) OR ("viral infection"[Title/Abstract])) OR ("viral infections"[Title/Abstract])) OR ("parasitic infection"[Title/Abstract])) OR ("parasitic infections"[Title/Abstract])) OR ("influenza"[Title/Abstract])) OR ("viral diseases"[Title/Abstract])) OR ("viral disease"[Title/Abstract])) OR ("herpesvirus infection"[Title/Abstract])) OR ("herpesvirus infections"[Title/Abstract])) OR ("fungus diseases"[Title/Abstract])) OR ("fungus disease"[Title/Abstract])) OR ("herpes simplex virus infection"[Title/Abstract])) OR ("herpes simplex virus infections"[Title/Abstract])) OR ("reactivated infection"[Title/Abstract])) OR ("reactivated infections"[Title/Abstract])) OR ("invasive mycoses"[Title/Abstract] OR "invasive mycosis"[Title/Abstract])) OR ("fungal diseases"[Title/Abstract])) OR ("fungal disease"[Title/Abstract])) OR ("fungus infections"[Title/Abstract])) OR ("fungus infection"[Title/Abstract])) OR ("toxicity"[Title/Abstract])) OR ("septicemia"[Title/Abstract])) OR ("toxicities"[Title/Abstract])))) OR ((((((((((((((((((((((((((((((((((((((((((((((((((((((((((((((((((((((((((("hematologic neoplasms"[Text Word]) OR ("hematologic neoplasm"[Text Word])) OR ("bone marrow neoplasms"[Text Word])) OR ("bone marrow neoplasm"[Text Word])) OR ("bone marrow failure disorders"[Text Word])) OR ("bone marrow failure disorder"[Text Word])) OR ("leukemia"[Text Word])) OR ("lymphoid leukemia"[Text Word])) OR ("myeloid leukemia"[Text Word])) OR ("acute myeloid leukemia"[Text Word]))) OR ("precursor cell lymphoblastic leukemia lymphoma"[Text Word])) OR ("bcr abl chronic myelogenous leukemia"[Text Word])) OR ("b cell chronic lymphocytic leukemia"[Text Word])) OR ("t cell leukemia"[Text Word])) OR ("acute biphenotypic leukemia"[Text Word])) OR ("lymphoma"[Text Word])) OR ("lymphomas"[Text Word])) OR ("non hodgkin lymphoma"[Text Word])) OR ("hodgkin disease"[Text Word])) OR ("t cell lymphoma"[Text Word])) OR ("b cell lymphoma"[Text Word])) OR ("marginal zone b cell lymphoma"[Text Word])) OR ("burkitt lymphoma"[Text Word])) OR ("peripheral t cell lymphoma"[Text Word])) OR ("anaplastic large cell lymphoma"[Text Word])) OR ("cutaneous t cell lymphoma"[Text Word])) OR ("diffuse large b cell lymphoma"[Text Word])) OR ("mantle cell lymphoma"[Text Word])) OR ("multiple myeloma"[Text Word])) OR ("plasma cell leukemia"[Text Word])) OR ("monoclonal gammopathy of undetermined significance"[Text Word])) OR ("smoldering multiple myeloma"[Text Word])) OR ("immunoglobulin light chain amyloidosis"[Text Word])) OR ("hematologic malignancies"[Text Word])) OR ("hematologic tumors"[Text Word])) OR ("hematological cancer"[Text Word])) OR ("hematological pathologies"[Text Word] OR "hematological pathology"[Text Word])) OR ("blood neoplasm"[Text Word])) OR ("blood neoplasms"[Text Word])) OR ("blood malignancies"[Text Word])) OR ("blood malignancy"[Text Word])) OR ("hemopoietic neoplasm"[Text Word])) OR ("hemopoietic neoplasms"[Text Word] OR "hemopoietic neoplastic"[Text Word])) OR ("bone marrow failure syndrome"[Text Word])) OR ("bone marrow failure syndromes"[Text Word])) OR ("acute leukemia"[Text Word])) OR ("chronic leukemia"[Text Word])) OR ("acute myeloid leukemia"[Text Word])) OR ("acute lymphocytic leukemia"[Text Word])) OR ("chronic myelogenous leukemia"[Text Word])) OR ("chronic myelogenous leukemias"[Text Word])) OR ("chronic lymphocytic leukemia"[Text Word])) OR ("b cell leukemia"[Text Word])) OR ("b cell acute lymphocytic leukemia"[Text Word])) OR ("t cell acute lymphoblastic leukemia"[Text Word])) OR ("t cell chronic lymphocytic leukemia"[Text Word])) OR ("hodgkin lymphoma"[Text Word])) OR ("marginal zone lymphoma"[Text Word])) OR ("nodular lymphocyte predominant hodgkin lymphoma"[Text Word])) OR ("extranodal lymphoma"[Text Word])) OR ("lymphoblastic lymphoma t cell"[Text Word])) OR ("t cell large cell lymphoma"[Text Word])) OR ("t cell peripheral lymphoma"[Text Word])) OR ("t cell extranodal lymphomas"[Text Word])) OR ("t cell extranodal lymphomas"[Text Word])) OR ("plasma cell myeloma"[Text Word])) OR ("light chain amyloidosis"[Text Word])) OR ("iga myeloma"[Text Word])) OR ("igg myeloma"[Text Word])) OR ("nonsecretory multiple myeloma"[Text Word])) OR ("follicular lymphoma"[Text Word])) OR ("b cell precursor acute lymphoblastic leukemia"[Text Word])) AND ((((((((((((((((((((((((((((((((((((("adoptive immunotherapy"[Text Word]) OR ("adoptive immunotherapies"[Text Word])) OR ("chimeric antigen receptors"[Text Word])) OR ("chimeric antigen receptor t cell"[Text Word])) OR ("chimeric t cell receptor"[Text Word])) OR ("chimeric t cell receptors"[Text Word])) OR ("chimeric immunoreceptor"[Text Word] OR "chimeric immunoreceptors"[Text Word])) OR ("chimeric antigen receptor therapy"[Text Word])) OR ("chimeric antigen receptor therapies"[Text Word])) OR ("tisagenlecleucel"[Text Word])) OR ("kymriah"[Text Word])) OR ("axicabtagene ciloleucel"[Text Word])) OR ("yescarta"[Text Word])) OR ("brexucabtagene autoleucel"[Text Word])) OR ("tecartus"[Text Word])) OR ("idecabtagene vicleucel"[Text Word])) OR ("abecma"[Text Word])) OR ("lisocabtagene maraleucel"[Text Word])) OR ("breyanzi"[Text Word])) OR ("ciltacabtagene"[Text Word])) OR ("ciltacabtagene autoleucel"[Text Word])) OR ("carvykti"[Text Word])) OR ("cd19 chimeric antigen receptor"[Text Word])) OR ("cd19 chimeric antigen receptors"[Text Word])) OR ("car t cell"[Text Word])) OR ("car t cells"[Text Word])) OR ("adoptive cellular immunotherapy"[Text Word])) OR ("adoptive cellular immunotherapies"[Text Word])) OR ("adoptive immunotherapy"[Text Word])) OR ("adoptive immunotherapies"[Text Word])) OR ("car t cell therapy"[Text Word])) OR ("car t cell therapies"[Text Word])) OR ("chimeric antigen receptor"[Text Word])) OR ("chimeric antigen receptors"[Text Word])) OR ("artificial t cell receptor"[Text Word] OR "artificial t cell receptors"[Text Word])) OR ("b cell maturation antigen"[Text Word])) OR ("cd19 antigens"[Text Word]))) AND ((((((((((((((((((((((((((((((((((((((((((((((((((((((((((((((((((((((((((((((((((((((((((("drug related side effects and adverse reactions"[Text Word]) OR ("infections"[Text Word])) OR ("infection"[Text Word])) OR ("febrile neutropenia"[Text Word])) OR ("cytomegalovirus infections"[Text Word])) OR ("cytomegalovirus infection"[Text Word])) OR ("virus diseases"[Text Word])) OR (Virus Diseases[Text Word])) OR ("virus disease"[Text Word])) OR ("bacterial infection"[Text Word])) OR ("bacterial infections"[Text Word])) OR ("fungi"[Text Word])) OR ("tuberculosis"[Text Word])) OR ("parasitic disease"[Text Word])) OR ("parasitic diseases"[Text Word])) OR ("sepsis"[Text Word])) OR ("abscess"[Text Word])) OR ("covid 19"[Text Word])) OR ("mycoses"[Text Word])) OR ("encephalomyelitis"[Text Word])) OR ("encephalitis"[Text Word])) OR ("meningoencephalitis"[Text Word])) OR ("myelitis"[Text Word])) OR ("opportunistic infections"[Text Word])) OR ("opportunistic infection"[Text Word])) OR ("respiratory tract infections"[Text Word])) OR ("respiratory tract infection"[Text Word])) OR ("pneumonia"[Text Word])) OR ("tracheitis"[Text Word])) OR ("bacteremia"[Text Word])) OR ("fungemia"[Text Word])) OR ("skin diseases"[Text Word])) OR ("skin disease"[Text Word])) OR ("soft tissue infections"[Text Word])) OR ("soft tissue infection"[Text Word])) OR ("suppuration"[Text Word])) OR ("urinary tract infections"[Text Word])) OR ("urinary tract infection"[Text Word])) OR ("hepatitis"[Text Word])) OR ("viremia"[Text Word])) OR ("human herpesvirus 1"[Text Word] OR "human herpesvirus 1 infection"[Text Word] OR "human herpesvirus 2"[Text Word] OR "human herpesvirus 3"[Text Word])) OR ("varicella zoster virus infection"[Text Word])) OR ("varicella zoster virus infections"[Text Word])) OR ("epstein barr virus infection"[Text Word])) OR ("epstein barr virus infections"[Text Word])) OR ("virus disease"[Text Word])) OR ("virus diseases"[Text Word])) OR ("herpesviridae infections"[Text Word])) OR ("herpesviridae infection"[Text Word])) OR ("drug related side effects"[Text Word])) OR ("drug related side effect"[Text Word])) OR ("adverse reactions included"[Text Word])) OR ("adverse drug reactions"[Text Word])) OR ("adverse drug reaction"[Text Word])) OR ("drug reactions"[Text Word])) OR ("adverse drug reaction"[Text Word])) OR ("drug toxicity"[Text Word])) OR ("drug toxicities"[Text Word])) OR ("adverse events"[Text Word])) OR ("adverse event"[Text Word])) OR ("pulmonary fungal diseases"[Text Word])) OR ("pulmonary fungal disease"[Text Word])) OR ("fungal infection"[Text Word])) OR ("fungal infections"[Text Word])) OR ("fungi infection"[Text Word])) OR ("fungi infections"[Text Word])) OR ("virus infection"[Text Word])) OR ("virus infections"[Text Word])) OR ("viral infection"[Text Word])) OR ("viral infections"[Text Word])) OR ("parasitic infection"[Text Word])) OR ("parasitic infections"[Text Word])) OR ("influenza"[Text Word])) OR ("viral diseases"[Text Word])) OR ("viral disease"[Text Word])) OR ("herpesvirus infection"[Text Word])) OR ("herpesvirus infections"[Text Word])) OR ("fungus diseases"[Text Word])) OR ("fungus disease"[Text Word])) OR ("herpes simplex virus infection"[Text Word])) OR ("herpes simplex virus infections"[Text Word])) OR ("reactivated infection"[Text Word])) OR ("reactivated infections"[Text Word])) OR ("invasive mycoses"[Text Word] OR "invasive mycosis"[Text Word])) OR ("fungal diseases"[Text Word])) OR ("fungal disease"[Text Word])) OR ("fungus infections"[Text Word])) OR ("fungus infection"[Text Word])) OR ("toxicity"[Text Word])) OR ("septicemia"[Text Word])) OR ("toxicities"[Text Word]))) – 3.231
